# Periodic ethanol supply as a path toward unlimited lifespan of *Caenorhabditis elegans* dauer larvae

**DOI:** 10.3389/fragi.2023.1031161

**Published:** 2023-09-05

**Authors:** Xingyu Zhang, Sider Penkov, Teymuras V. Kurzchalia, Vasily Zaburdaev

**Affiliations:** ^1^ Friedrich-Alexander-Universität Erlangen-Nürnberg, Erlangen, Germany; ^2^ Max-Planck-Zentrum für Physik und Medizin, Erlangen, Germany; ^3^ Center of Membrane Biochemistry and Lipid Research, University Clinic and Faculty of Medicine, Dresden, Germany; ^4^ Institute for Clinical Chemistry and Laboratory Medicine, University Clinic and Faculty of Medicine, Dresden, Germany; ^5^ Max Planck Institute of Molecular Cell Biology and Genetics, Dresden, Germany

**Keywords:** dauer larvae, lifespan extension, metabolic network, mathematical model, ethanol, periodic feeding

## Abstract

The dauer larva is a specialized stage of worm development optimized for survival under harsh conditions that have been used as a model for stress resistance, metabolic adaptations, and longevity. Recent findings suggest that the dauer larva of *Caenorhabditis elegans* may utilize external ethanol as an energy source to extend their lifespan. It was shown that while ethanol may serve as an effectively infinite source of energy, some toxic compounds accumulating as byproducts of its metabolism may lead to the damage of mitochondria and thus limit the lifespan of larvae. A minimal mathematical model was proposed to explain the connection between the lifespan of a dauer larva and its ethanol metabolism. To explore theoretically if it is possible to extend even further the lifespan of dauer larvae, we incorporated two natural mechanisms describing the recovery of damaged mitochondria and elimination of toxic compounds, which were previously omitted in the model. Numerical simulations of the revised model suggested that while the ethanol concentration is constant, the lifespan still stays limited. However, if ethanol is supplied periodically, with a suitable frequency and amplitude, the dauer could survive as long as we observe the system. Analytical methods further help to explain how feeding frequency and amplitude affect lifespan extension. Based on the comparison of the model with experimental data for fixed ethanol concentration, we proposed the range of feeding protocols that could lead to even longer dauer survival and it can be tested experimentally.

## 1 Introduction

**FIGURE 1:**
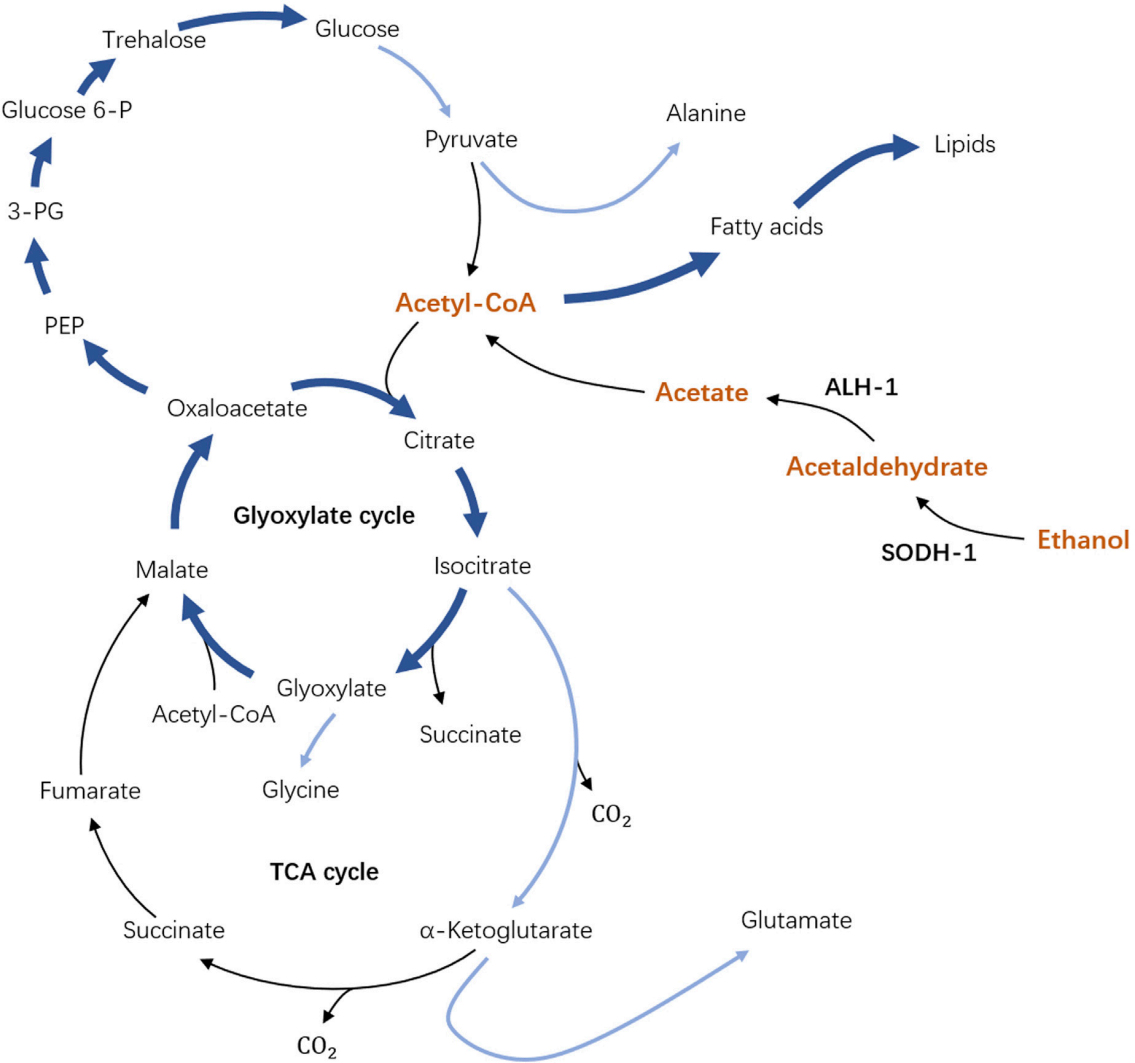
Schematics of the metabolic pathway of dauer larvae in the presence of external ethanol. In dependence on SODH-1 and ALH-1 enzymes, external ethanol is broken up and activated to Acetyl-COA (part of the pathway shown in orange) which is the key element of the original unperturbed metabolic pathway. Importantly, the arrows now pointing from Acetyl-CoA to fatty acids show the possibility of lipid storage accumulation in the presence of ethanol.

*Caenorhabditis elegans* is a well-known free-living nematode studied as a model organism to address a broad range of biomedical questions from genetics, cell biology, and human disease conditions to nematode control Sydney (1974); Corsi et al. (2015). In the context of how organisms may adapt to stressful environmental conditions, *C. elegans* larval stage called “dauer” is of particular interest Riddle (1988); Hu (2007); Erkut and Kurzchalia (2015). A developing *C. elegans* larva at the L1 stage can turn into an alternative dauer larva developmental stage under harsh environments such as lack of food or high population density Riddle (1988); Hu (2007); Erkut and Kurzchalia (2015). To be able to survive these conditions, *C. elegans* dauer develop a strong cuticle that covers its whole body, such that most of the matter exchange across its body boundary shuts down. As a result, it was long believed that *C. elegans* dauer survive solely on stored lipids and are not able to uptake any carbon source from their environment Riddle (1988); Hu (2007); Erkut and Kurzchalia (2015). However, our recent findings Kaptan et al. (2020) showed that *C. elegans* dauer can utilize ethanol as an external carbon source, see Figure 1. Remarkably, at optimal concentrations, ethanol could expand the lifespan of dauer larvae twofold for a wild type and up to fourfold for some mutants. Ethanol can penetrate across the cuticle and thus gets channeled in the metabolic pathways of *C. elegans* dauer larvae. The enzymes responsible for the first metabolic steps are SODH-1 and ALH-1 which transform ethanol to acetate which can be activated into acetyl-COA and enters the major metabolic pathways of the TCA cycle, glyoxylate shunt, gluconeogenesis, and lipid metabolism, thus augmenting the metabolic pathways that dauers use for energy production Kaptan et al. (2020); Erkut and Kurzchalia (2015); Penkov et al. (2020); Burnell et al. (2005); Wadsworth and Riddle (1989). SODH-1 and ALH-1 are found to be upregulated in the presence of ethanol, whereas in *sodh-1* mutant, the ethanol is no longer incorporated and does not affect the lifespan of dauer. Experiments with radioactively-labeled ethanol have shown that it can be utilized for the production and accumulation of stored lipids, thus providing an effectively unlimited source of energy to dauer larvae in case of permanent ethanol supply Kaptan et al. (2020).

This led us to the question of why even in the presence of this energy source dauers do exhibit a longer lifespan but then eventually die. To help to answer this question, we proposed a mathematical model describing the relation between the lifespan of *Caenorhabditis elegans* dauer and the supplied ethanol based on the known metabolic pathway of dauer larvae. We assumed that the dauer dies either due to the lack of energy or due to the accumulation of some not yet identified toxic compound(s) Kaptan et al. (2020) that could resemble the so-called “lipotoxicity” factors in mammalian systems Burnell et al. (2005); Schooneman et al. (2013); DeFronzo (2010). As experimentally observed, the death of worms was preceded by the deterioration of mitochondria. We also assumed that these two mechanisms led to mitochondria damage and then to death. This model was very successful in explaining experimental data on the lifespans of dauer and various mutants in the presence of and without ethanol.

While identifying the exact toxic component that limits the lifespan of dauer is still an ongoing research project, we were interested in exploring whether or not the lifespan could be extended even further. To this end, we assume there are two self-recovery mechanisms, namely, regeneration of mitochondria and detoxification, and we test what they lead to. These two mechanisms alone still result in dauer’s death if the feeding protocol is constant. However, when we use a periodic supply of ethanol in the model, an unlimited lifespan can emerge according to the numerical simulation. By comparing model predictions with existing data on constant feeding, we also suggest feeding protocols that can be directly tested in future experiments on dauer.

Certainly, the unlimited lifespan for the wild-type *C. elegans* dauer larvae even under the proposed feeding protocol sounds unfeasible and is a regime predicted by an idealized theoretical model that proposes two recovery mechanisms. However, testing this model prediction in experiments will provide crucial insights into the metabolism of dauers if there is *any* significant extension of the lifespan for periodic feeding that would support the hypothesis of the existence of recovery mechanisms that could be further explored.

## 2 Methods

### 2.1 Mathematical model

A minimal model of the metabolic network of *C. elegans* dauer larvae was introduced in Kaptan et al. (2020) and accurately reproduced the lifespans of dauer with and without ethanol for wild-type worms and various mutations. The framework of the model follows the largely coarse-grained metabolic pathway of dauer. All the chemical components falling into the category of “available energy” are combined and called “acetate”, which is the central representative component of this category. Similarly, the components corresponding to “stored energy” and “consumed energy” are denoted as lipids and carbohydrates, respectively. Acetate and lipids could transform into each other as the balance between free and stored energy. At the same time, acetate continuously transforms into carbohydrates unidirectionally to support the main functions of an organism including mitochondria. If the production of carbohydrates drops below a certain minimal threshold, mitochondria start to get damaged and the dauer dies. In the presence of ethanol, acetate gains an influx proportional to its concentration. During the process of releasing stored lipids, toxic compounds are produced as a side product and as the second major reason that causes damage to the mitochondria alongside the lack of carbohydrate production.

Our model also included the effect of genes that were identified as regulatory factors through genetic experiments with loss-of- or reduction-of-function mutations Kaptan et al. (2020). We use *daf-2(e1370)* strain as a proxy for the control strain in our current study. The differences between wild-type dauers and *daf-2* mutants were previously discussed in Kaptan et al. (2020). Specifically, this strain has a Daf-c (dauer constitutive) phenotype due to a conditional, temperature-activated mutation that induces dauer formation even on ample food and at a low population density. It undergoes controlled dauer formation upon temperature switch and provides two advantages compared to the wild-type: i) *daf-2* animals enter dauer state more synchronously and, thus, the age variations are minor; ii) as they enter dauer state under unrestricted food supply, they might be able to store more lipids prior to entering dauer arrest. Otherwise, there is a well-documented agreement in the field that *daf-2* dauer larvae recapitulate to a high degree the signaling and metabolic processes in wild-type worms undergoing dauer formation Hu (2007). Loss-of-function mutation in the *aak-2*/AMPKα in *daf-2(e1370);aak-2(gt33)* double mutants causes an enhanced lipolysis rate, which leads to a reduced lifespan compared to the control strain under both feeding conditions with and without ethanol Penkov et al. (2020); Narbonne and Roy (2009). A reduction-of-function allele in the class I PI3-kinase *age-1(hx546)* (single mutant), on the other hand, is supposed to have a reduced lipid synthesis rate. This assumption is based on experimental results with the mutant *age-1* in dauer state Kaptan et al. (2020). Its lifespan is similar to control dauer when there is no ethanol supply but has a large increase when the ethanol is supplied Kaptan et al. (2020).

**FIGURE 2:**
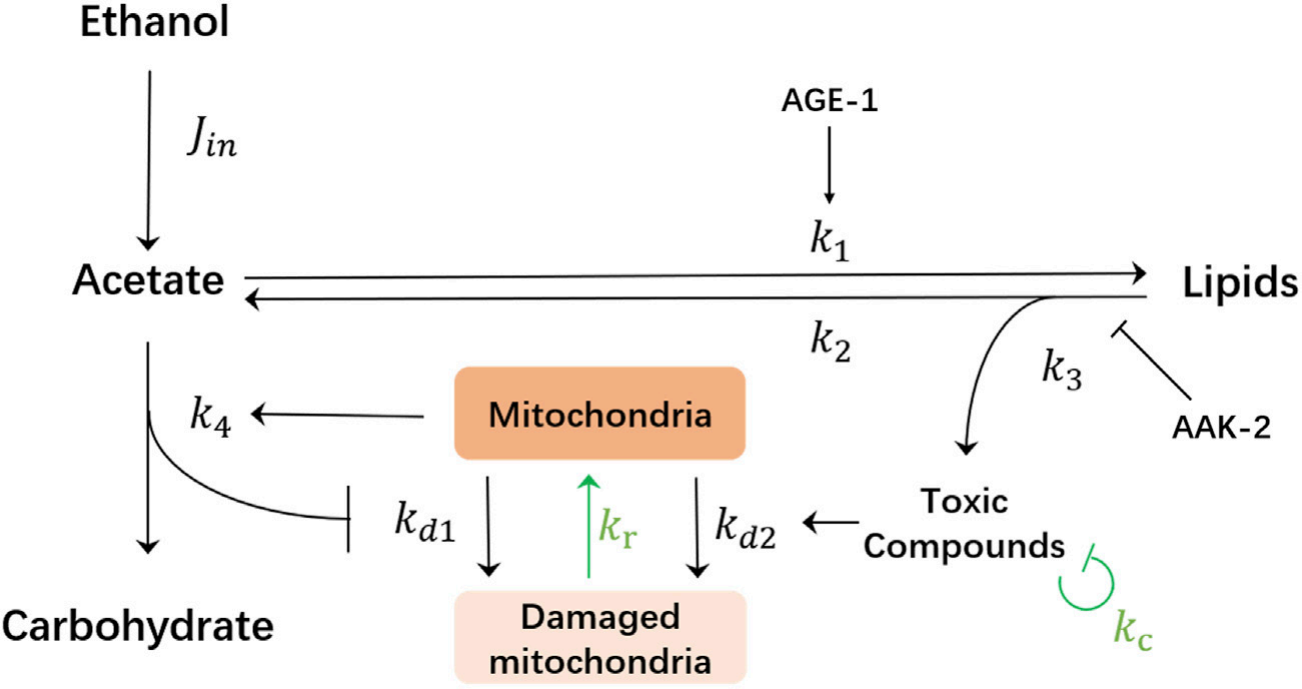
Schematics of the mathematical model of the metabolic network of *C. elegans* dauer larvae. Externally supplied ethanol is transformed into the acetate creating its influx *j*_in_, which can be either constant or varies in time depending on the respective supplied ethanol concentration. Acetate (ch. acetyl-CoA in Figure 1) is then used either for energy production and carbohydrate synthesis or can be stored in lipids. Mitochondria undergo damage from lack of carbohydrate production or accumulation of toxic compounds as the product of lipolysis. Aak-2 kinase exerts the inhibitory effect on the lipolysis, thus the *aak-2* mutation leads to an enhanced lipolysis rate. Age-1 kinase stimulates lipid synthesis and thus *age-1* mutation would lead to the reduction of lipid synthesis. Green arrows indicate two hypothesized mechanisms of recovery by degradation of toxic compounds with the rate *k*_c_ and mitochondria regeneration with the rate *k*_r_.

The goal of this work is to identify potential ways of how the dauer could survive even for a longer time. Thus, here, we consider mechanisms by which the model will be able to produce an unlimited lifespan while still remaining consistent with the results of the previous experiments. There are two essential and rather natural mechanisms that have been omitted in the original model Kaptan et al. (2020) while having potential for lifespan extension: detoxification Xu et al. (2005); Chen (2011) and a possibility for mitochondria to regenerate Palikaras and Tavernarakis (2014); Ni et al. (2015); Chan (2012) (see green arrows in Figure 2). We will show in the following that the model containing these two mechanisms predicts the possibility for lifespan extension under periodic supply protocol of ethanol.

To demonstrate this, we first formalize the schematics in Figure 2 into the system of ordinary differential equations that describe the chemical reaction network of ethanol metabolism Fromm and Hargrove (2011):

$$\frac{da}{dt}=-\left(k_{1}+k_{4}\right)a+k_{2}l+j_{in},$$ (1)

$$\frac{dl}{dt}=k_{1}a-k_{2}l,$$ (2)

$$\frac{dc}{dt}=k_{3}k_{2}l-k_{c}c,$$ (3)

$$\begin{array}{cc} \frac{dm}{dt} & =-k_{\text{d}1}\Theta \left(k_{4}a-j_{m}\right)m-k_{\text{d}2}\Theta \left(c_{h}-c\right)m\\ & \;+k_{r}\left(1-\Theta \left(k_{4}a-j_{m}\right)\right)\left(1-\Theta \left(c_{h}-c\right)\right)\left(1-m\right). \end{array}$$ (4)

Here “*a*” and “*l*” denote the concentrations of the acetate and lipids, respectively, while “*c*” represents the concentration of toxic compound(s), and *m* designates the wellbeing of mitochondria. The consumption of acetate for simplicity is assumed to be unidirectional (not explicitly modeled in the system) with the rate *k*_4_, but acetate can also be stored in lipids, see Eq. 1. In the presence of external ethanol, an influx *j*_in_ of acetate is included as the source. This influx *j*_in_ is assumed to be proportional to the external ethanol concentration. Lipids get created from acetate with rate *k*_1_ while they are released through the lipolysis process with rate *k*_2_, Eq. 2. The toxic compound(s) *c* is produced as the side product of lipolysis with proportionality factor *k*_3_ and spontaneous degradation rate *k*_c_, Eq. 3. The variable *m* ranges from 1 to 0 and denotes the wellbeing of mitochondria, where *m* = 1 means a fully functional mitochondria and *m* = 0 means a fully damaged one. Mitochondria can be damaged with a rate *k*_d1_ if the carbohydrate production *k*_4_ *a* falls below the minimal required “energy” flux *j*_m_, or with a rate *k*_d2_ when the toxic compound *c* accumulates above a certain threshold concentration *c*_h_ (Θ in the equation is the Heaviside step function). There are many known mechanisms of mitochondria surveillance and maintenance Palikaras and Tavernarakis (2014); Ni et al. (2015); Chan (2012). Here, for simplicity, we suggest a phenomenological law of mitochondria recovery. When mitochondria do not suffer any damage (i.e., there is enough ethanol and the toxic compound is below the critical threshold) they can regenerate. The regeneration rate is proportional to the current damage level of mitochondria (1 − *m*) with a rate constant *k*_r_. This term ensures that the value of *m* recovers toward one from any state of 0 < *m* < 1.

While most of the reaction rates in the above equations are considered constant for simplicity, some rates do depend on variables. The first example is the linear dependence of *k*_4_ on *m*, which assumes that the energy production requires functional mitochondria:

$$k_{4}={\tilde{k}}_{4}m,$$ (5)

where ${\tilde{k}}_{4}$ is a constant. Another non-constant rate is *k*_1_ quantifying acetate-to-lipids conversion. It reflects the fact that each dauer has a storage limit capacity *l*_s_ (it cannot accumulate unlimited amounts of lipids):

$$k_{1}={\tilde{k}}_{1}\frac{l_{s}-l}{l_{1}+\left(l_{s}-l\right)}.$$ (6)

Here, *l*_1_ is a characteristic lipid concentration at which the conversion starts to saturate and ${\tilde{k}}_{1}$ is a constant. Finally, we also assume that *k*_2_ has the functional form of Michaelis-Menten reaction Fromm and Hargrove (2011).

$$k_{2}={\tilde{k}}_{2}\frac{1}{l_{2}+l},$$ (7)

where ${\tilde{k}}_{2}$ and *l*_2_ are constants. If in the above equations, we set *k*_*r*_ = 0 and *k*_*c*_ = 0, we will recover the system studied in Kaptan et al. (2020).

The model including the self-recovery mechanism, however, should also reproduce the lifespan of dauers with and without ethanol, as well as different mutants as was observed experimentally Kaptan et al. (2020). This also means that this model should result in a finite lifespan under a constant ethanol supply. However, the novel possibility for lifespan extension may now emerge for non-constant feeding, where the supplied ethanol concentration varies in time, for instance, according to a periodic sinusoidal protocol. We next show by using numerical simulations that the model reproduces experimental observations under constant feeding and predicts the lifespan extension under periodic feeding protocol.

## 3 Results

### 3.1 Constant ethanol supply

**FIGURE 3:**
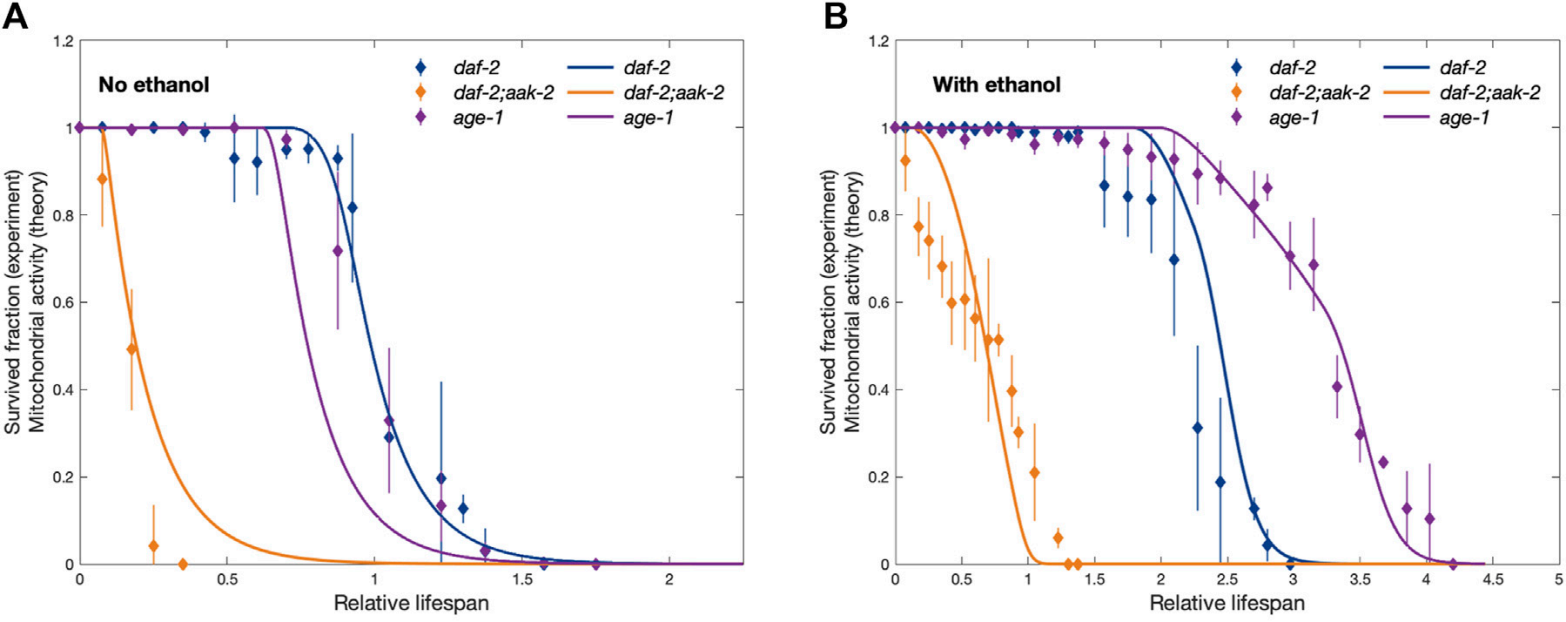
Comparison between experimentally observed lifespan of dauers of different strains (dashed lines) showing the fraction of survived dauers as a function of time and the corresponding simulation results (solid lines) showing the activity of mitochondria. Panels **(A)** and **(B)** correspond to no ethanol and provided ethanol conditions, respectively. The time axis is rescaled for the experimental curves by the time at which the fraction of survived *daf-2* strain dauers used as a control drops to 0.5 (50%) in no ethanol condition (*t* =40 days). Similarly, for the simulation results, the time axis is rescaled by the time at which the activity of mitochondria in control conditions with no ethanol drops to 50%. This rescaling allows for direct theory-experiment comparison even if we do not link simulation time to real-time units. Experimental data is taken from Kaptan et al. (2020), and error bars show standard deviation.

We first check if the model with the self-recovery can recapitulate experimental observations with a constant ethanol supply. Parameters used in the simulations were chosen by checking whether the lifespan ratios between mutants (*daf-2*, *daf-2;aak-2*, and *age-1)* with and without ethanol generated by simulations fit the previous experimental results Kaptan et al. (2020). When one set of parameters is considered the control strain without ethanol feeding, the corresponding parameter set of *daf-2;aak-2* mutant is defined by increasing the value of ${\tilde{k}}_{2}$ while keeping other parameters unchanged. Similarly, *age-1* mutant has a reduced ${\tilde{k}}_{1}$ constant. Three strains under two feeding conditions give rise to a total of six sets of parameters including the starved control strain as the baseline parameter set. We can call these sets a parameter collection. A parameter collection of the model is said to reproduce the experimental observation if all the ratios of lifespans produced by its parameter sets reproduced the experimentally observed values. Figure 3 shows the reproduction of the observed lifespan ratios by the model with self-recovery.

**FIGURE 4:**
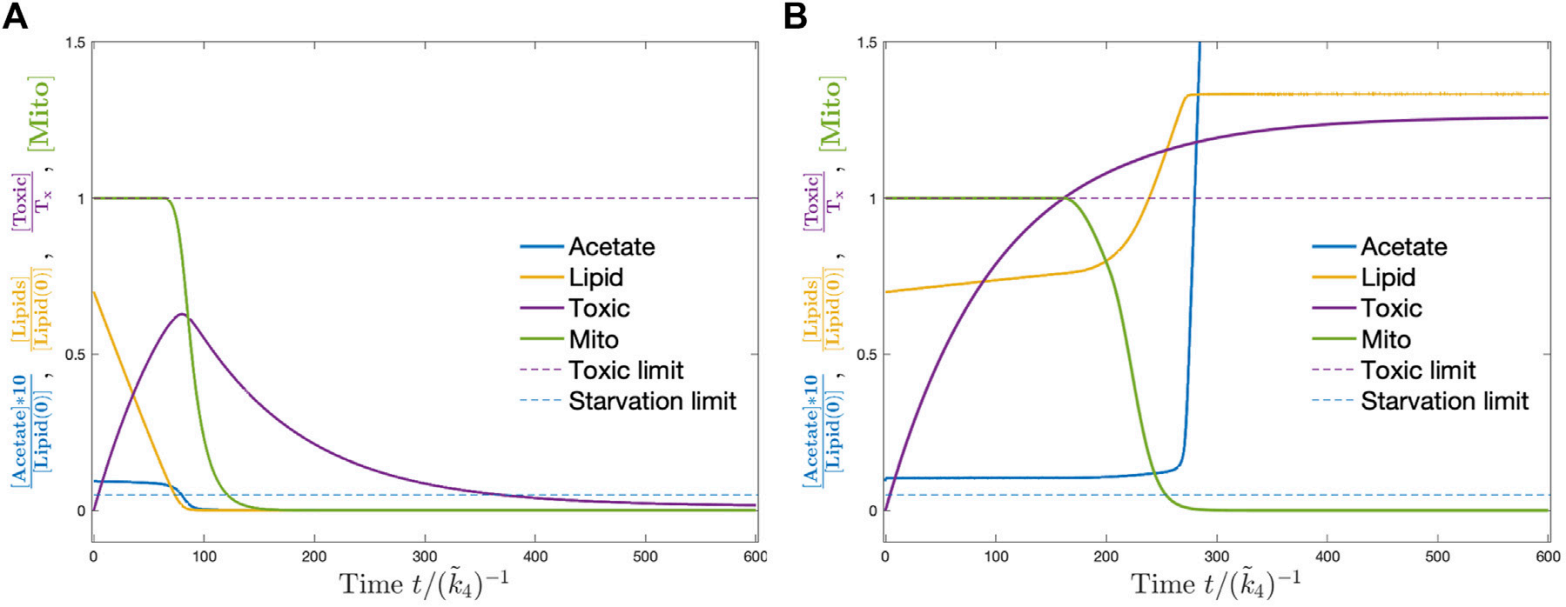
Dynamics of the model corresponding to control strain without **(A)** and with ethanol **(B)**. Without ethanol supply, the dauer consumes the stored lipids (yellow) which keep acetate levels (blue) and mitochondria (green) at constant levels. Toxic compounds accumulate (magenta). When lipids are used up, the acetate level drops below the critical threshold (dashed blue line) and mitochondria start to get damaged till the larvae die due to starvation. With ethanol supply, acetate never ends and mitochondria start to damage after the toxic compounds go above a critical threshold (dashed magenta line). Then, dauer dies due to the accumulation of toxic compounds.

The detailed dynamics of one of the parameter sets for control strain with and without feeding is shown as an example in Figure 4. When there is no feeding, dauer breaks down storage lipids to keep its acetate level and thus the carbohydrate production rate. As the lipids run out, the mitochondria are damaged for lack of carbohydrate production which results in the death of dauer. When ethanol is supplied at a sufficient level, starvation no longer becomes a concern. However, the toxic compound continuously accumulates and as it goes beyond the threshold at some point, the mitochondria start to take damage and finally the larvae die. The details of the simulation including the numerical methods Fromm and Hargrove (2011); Murray (1989); Strogatz (2000) and parameters are provided in Supplementary Appendix S1.

As the above two examples show, starvation or accumulation of toxic compounds is the reason for mitochondria damage and the resulting death of larvae. We can demonstrate more generally the condition for the finite lifespan of dauers for a constant ethanol concentration.

**FIGURE 5:**
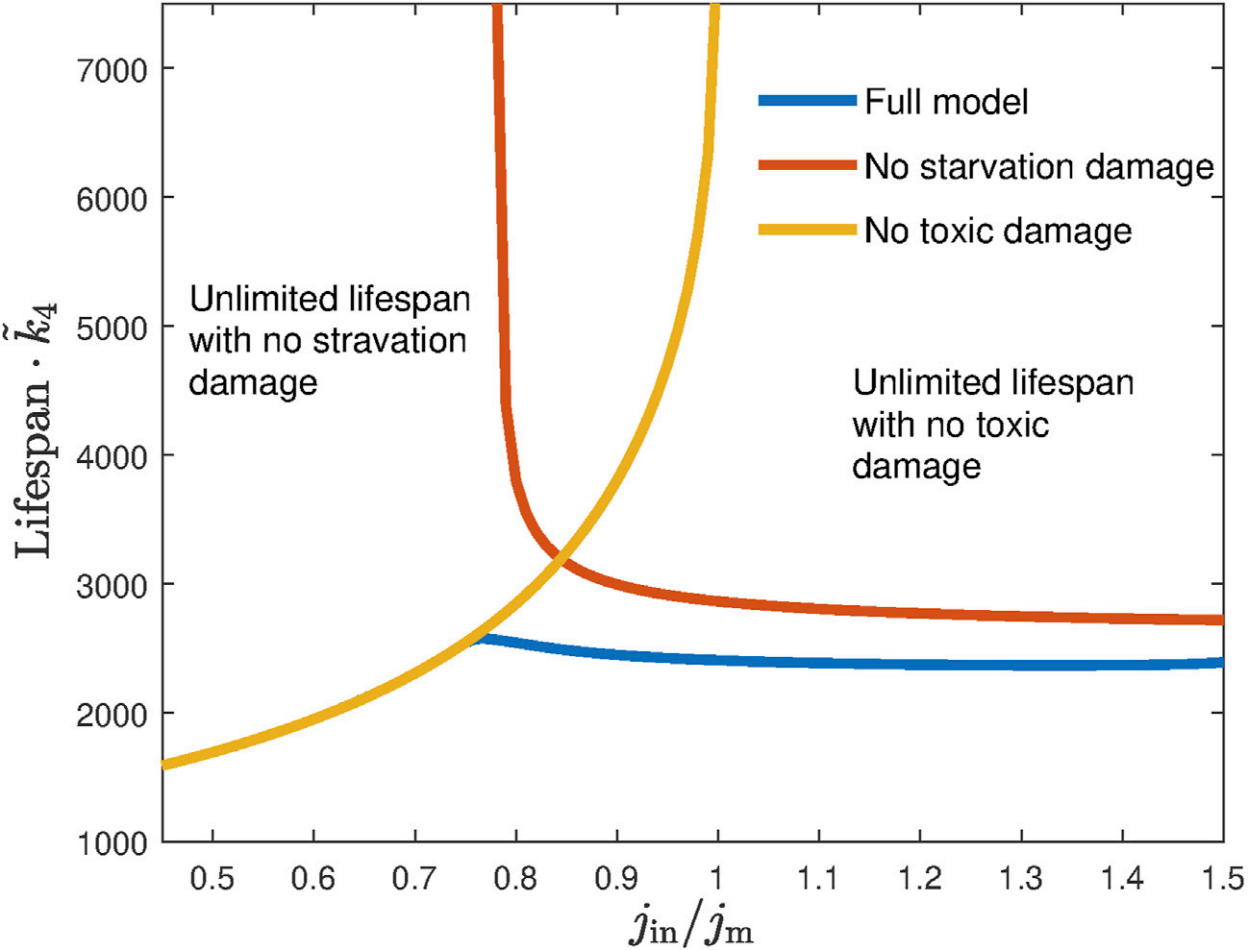
No lifespan extension is possible when larvae are fed with constant ethanol influx. The red and yellow lines show the lifespan as a function of the ethanol influx (normalized by minimal energy flux *j*_m_ required for mitochondria wellbeing) in the absence of toxic damage and no starvation damage, respectively. While both cases have regions of influx supporting infinite lifespan, they do not overlap, leading to the overall final lifespan when both damages are present (blue line).

Figure 5 shows the lifespan of dauer as a function of the external ethanol influx. If either starvation damage *k*_d1_ or toxic damage *k*_d2_ is removed, the dauer may have an infinite lifespan when the ethanol concentration is sufficiently high or low, respectively. If, however, these two lifespans vs. influx curves intersect at some value of *j*_in_, lifespan will always remain finite, and as for any given ethanol concentration and the corresponding influx, there will be at least one reason that the dauer dies within the limited time determined by *k*_*d*1_ or *k*_*d*2_.

If the two curves (with each of the damage removed) do not intersect, they would form boundaries of a domain in between where the value of *j*_in_ would support an infinite lifespan. As we mentioned above, in experiments, the dauer always survives the finite time in the presence of ethanol, thus defining for us the parameter range that has to be chosen in simulations.

### 3.2 Periodic ethanol supply

The above results show that ethanol supply keeps mitochondria operational, but the accumulating toxic compounds damage the mitochondria. Here, we hypothesize that periodic ethanol supply might be the key to an unlimited lifespan of dauer. While periods of supply might be used to replenish lipid storage and repair mitochondria, periods of no feeding can be used to degrade the accumulated toxic compounds. We now test this hypothesis numerically. For simplicity, we use a sinusoidal feeding protocol with a feeding amplitude *A*, feeding frequency *ω*_*E*_, and a positive baseline value *j*_0_:

$$j_{\text{in}}=j_{0}+A\sin\left(\omega_{E}t+\phi \right).$$ (8)

With a proper parameter choice, the numerical simulations of the model show that the mitochondria damage and regenerate periodically until the end of simulations no matter how long these last.

**FIGURE 6:**
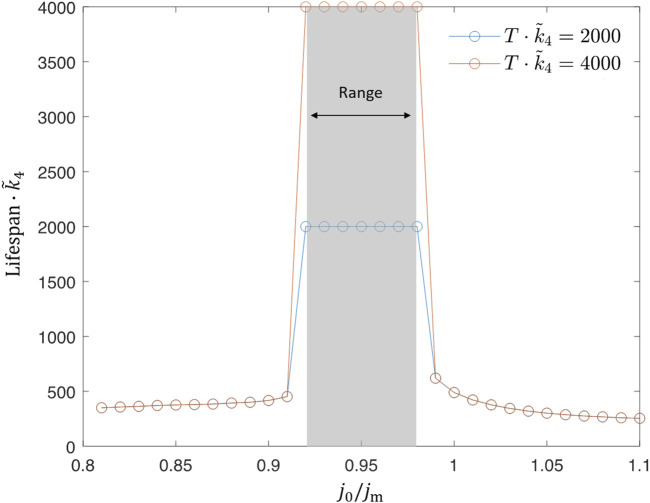
Example of a lifespan extension as a result of periodic feeding. The baseline value *j*_0_/*j*_m_ =0.8 while *A* = *j*_0_. The feeding frequency *ω*_*E*_ is the same as the oscillatory frequency of acetate level *a* shown in blue. The inset shows oscillations of toxicity near but below the toxic limit, while mitochondria are almost fully active.

Indeed, this situation becomes possible when parameters are tuned such that the periodic feeding permits the worm to accumulate toxic compounds while intaking ethanol and fuelling mitochondria but then remove them with a diet at the cost of some mitochondria damage, which can, however, be regenerated during the next intake cycle. These simulations suggest that the periodic feeding protocol does provide a theoretical possibility of an unlimited lifespan extension (Figure 6). We next investigate in more detail how this effect depends on model parameters.

Numerically, an unlimited lifespan can be defined as survival until the end of the simulation regardless of the simulation time. However, in practice, the time for which we can observe the system is always limited. Thus, we set a certain threshold value *T*_max_ for the survival time. If a dauer survives until *T*_max_ in simulation, we say the lifespan of the dauer is unlimited under this set of parameters. Our analytical considerations also suggest that there may exist a true infinite lifespan given a certain set of parameters in the model.

### 3.3 Effect of feeding parameters

**FIGURE 7:**
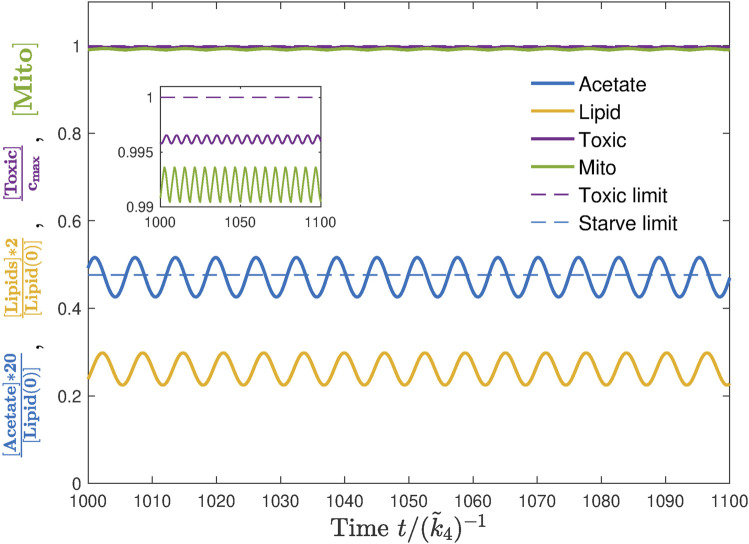
Range *w* is the size of an interval of *j*_0_ where the dauer experiences lifespan extension. Its value is obtained numerically by scanning over the baseline value *j*_0_ while keeping *ω*_*E*_ and *A* fixed. For each *j*_0_, we run multiple simulations corresponding to various initial lipid storage *l*(0) and take the largest *w* from those simulations as the range *w* corresponding to this *j*_0_. The baseline value *j*_0_ can be smaller than the minimal energy flux *j*_m_ as the oscillations with large enough amplitude *A* make *j*_in_ larger than *j*_m_ for a certain fraction of the feeding period. Here, we also show that the range does not strongly depend on the chosen threshold, $T_{\text{max}}\cdot {\tilde{k}}_{4}-2000,4000$ , which is approximately 20 and 40 times greater than the survival time of control strain without ethanol.

A single simulation does not reflect the whole picture but only indicates a possibility. To quantify the robustness of lifespan extension, we defined a new value “range” *w* as the size of the interval within which the baseline influx *j*_0_ may vary so that the dauer exhibits an unlimited lifespan, see Figure 7. The range *w* of this baseline interval thus quantifies the ability of a certain set of parameters (*ω*_*E*_, *A*) to support the extension of lifespan. We note that the lifespan seems to go through a sharp transition from a finite to an infinite value, first when approaching from the side of low baseline value and second when approaching from the side of large baseline levels. The transition at low influx seems to be a jump-like switch (as we could only test numerically). The high influx condition is amenable to analytical analysis and we could show that it has a shape of a logarithmic divergence (see Supplementary Appendix S3). By quantifying the survival ability with the value of *w* and studying its dependence on feeding parameters, *ω*_*E*_ and *A* would eventually help us to identify the optimal experimental conditions where the lifespan extension of dauer could be tested.

**FIGURE 8:**
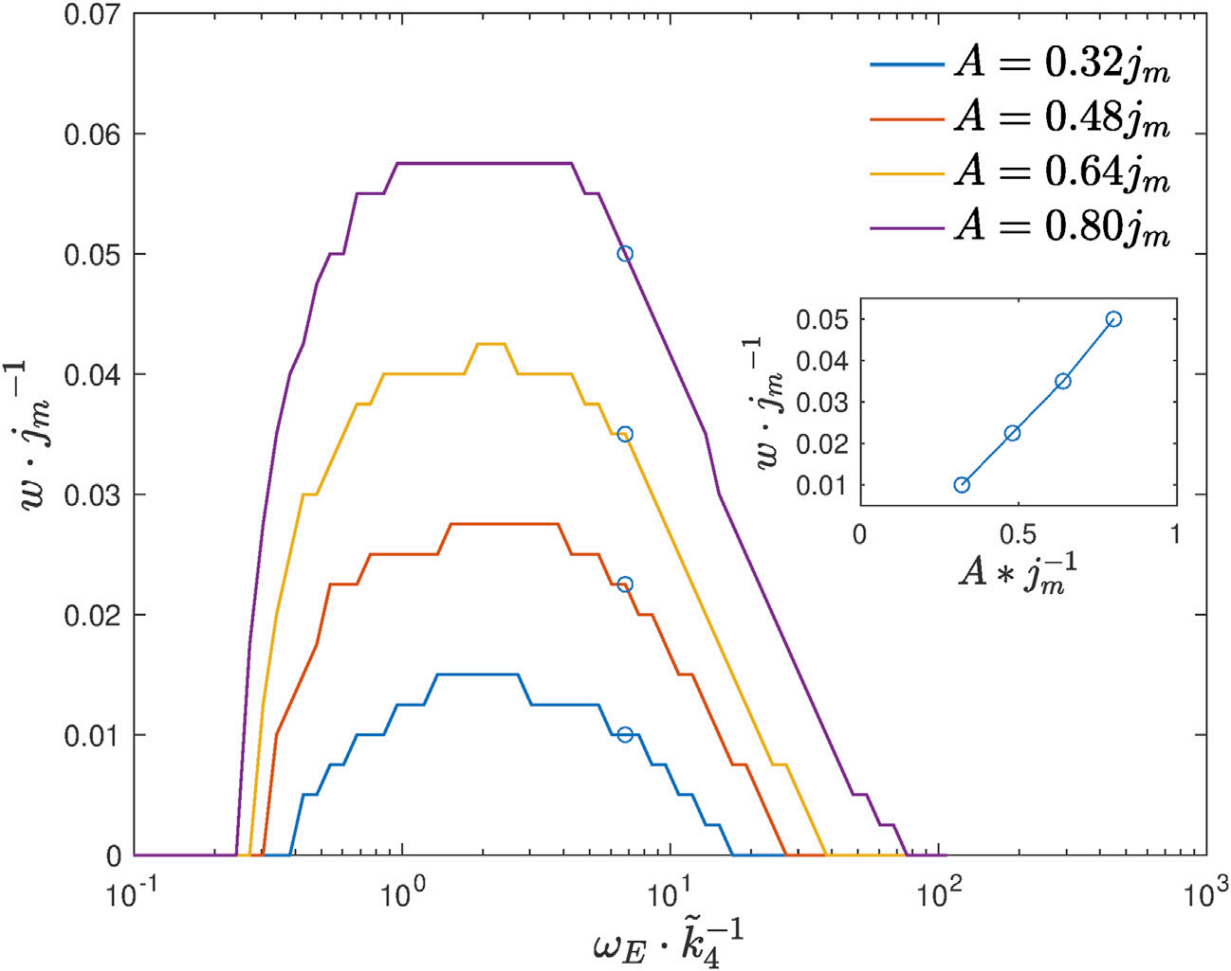
Range *w* as a function of the feeding frequency *ω*_*E*_ for different values of amplitude *A*. The inset shows a linear-like dependence of the range on amplitude in the region of high-feeding frequencies.

We next plot the range *w* as a function of feeding amplitude *A* and frequency *ω*_*E*_, see Figure 8.

According to the simulation results, we see that lifespan extension is possible when the feeding frequency is within a certain interval. The experiment with the feeding frequency *ω*_*E*_ corresponding to the maximal range *w* is expected to give the highest chance to observe the effect. The simulations also suggest that the range *w* grows with feeding amplitude *A* in an almost linear way if the feeding frequency is high enough. This is because, at a high-frequency region, the range *w* is proportional to the oscillation amplitude of acetate, which can be explained by an approximate analytical solution. (For details of the analysis, see Supplementary Appendix S2).

**FIGURE 9:**
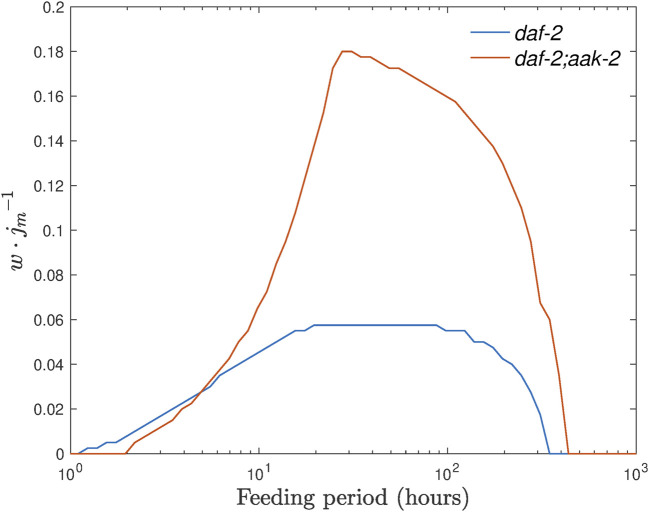
Range of the baseline ethanol supply levels where we expect to see unlimited survival of dauer larvae shown for control and *daf-2;aak-2* strains as a function of the feeding period *T* =2*π*/*ω*_*E*_. Here, it is assumed that the amplitude *A* takes the value of min[*j*_0_].

The range-frequency curves can potentially help us to identify suitable feeding frequencies and amplitudes for which it is most likely to observe lifespan extension in experiments. To do so, we still need to connect our mostly dimensionless equations to realistic parameters. This is not too straightforward since not all parameters of the enzymatic kinetics as well as chemical concentrations in the dauer were measured yet. However, for the case of the feeding frequency, we may take a short-cut where we can determine the timescale by equalling the control lifespan without ethanol in the model defined as the time where *m* falls to, for example, 0.5, compared to that in experiment defined as respective 50% survival and restore all reaction rates in real-time units. Also, the feeding amplitude is simply set as large as possible (see below) so there is no more information needed. Figure 9 shows the range vs. period (given in hours) relation for control strain and *daf-2;aak-2* mutants under maximal feeding amplitude. The maximal feeding amplitude is defined as *A* = min[*j*_0_] (i.e., the smallest *j*_0_ among all *j*_0_ used in scanning, such that the influx *j*_in_ is always positive). Another definition *A* = *j*_0_ for all *j*_0_ is also possible and leads to similar results.

These simulations not only suggest an optimal feeding period for both strains but also indicate that *daf-2;aak-2* mutant is a better option for the experiment, not only for the larger range value but also for the smaller optimal feeding period. It requires a feeding period of the order of 10 h (so the media for larvae can be changed once a day) and the effect should be seen much earlier, as the original lifespan of *daf-2;aak-2* is much shorter, and thus overall a shorter experiment could be carried out.

## 4 Discussion and conclusion

Previously, we have shown that the lifespan of *Caenorhabditis elegans* dauer larvae can be greatly extended due to the metabolism of externally provided ethanol. With the help of the mathematical model of this metabolic pathway, we proposed that the lifespan remains limited due to the accumulation of toxic compounds resulting from the process of lipolysis. So far, however, we have neglected the possibility of mechanisms that help dauer to recover from this damage. Therefore, two biological self-recovery mechanisms, namely, detoxification and mitochondria regeneration, were introduced into the model. Importantly, despite self-recovery mechanisms for constant ethanol supply, the model reproduces the experimental observations of extended but limited lifespan.

However, when the feeding protocol is periodic, an unlimited lifespan can emerge. The possibility of an unlimited lifespan can be explained by the switch between two feeding phases, where the first one at high ethanol concentration repairs the mitochondria at the cost of toxic compounds’ accumulation, while the second one, at low ethanol concentration, has the toxic compounds degraded but also damages the mitochondria slightly. For this process to keep the dauer surviving, both mitochondria regeneration and toxic compounds detoxification mechanisms are required to function.

Specifically in the context of dauer larvae survival in the wild Schulenburg and Félix (2017); Frézal and Félix (2015); Félix and Duveau (2012), our results might indicate that the time-varying availability of ethanol supply (when feeding phases are interspersed with starvation and toxic compound degradation) might be more beneficial for lifetime extension. One could speculate that fluctuating environmental conditions might be more common than a steady and abundant ethanol supply.

To characterize the unlimited lifespan predicted by the model systematically, we defined a range of baseline feeding fluxes, which quantifies the ability of a certain set of feeding parameters to support the unlimited lifespan. The dependence of this range on feeding frequency and amplitude was studied numerically with some supporting analytical arguments. This dependence combined with previous data helped us to suggest a suitable feeding period and amplitude that can now be tested experimentally.

While an unlimited lifespan is not uncommon in nature and is exemplified in worms by some planarian flatworm species Tan et al. (2012), observing this for the short-lived wild-type *C. elegans* dauer larvae is not likely. Our model, proposed and experimentally tested for constant ethanol supply, suggests that under the assumption of the existence of two recovery mechanisms (mitochondria repair and degradation of toxins) in a rather broad range of parameters, an unlimited lifespan is theoretically possible. Any significant extension of the lifespan for periodic feeding in the experiment would support the hypothesis of the existence of recovery mechanisms. Furthermore, an extended but limited lifespan would trigger the search for other yet unknown mechanisms that serve as limiting factors. Importantly, it also highlights the importance of ultimately identifying candidates of toxic compounds which, in the model, are the main cause of death when starvation is overcome by ethanol. This study treats the identity of toxic compounds openly and does not specify the concrete mechanisms of mitochondria recovery and detoxification. Ultimately, for our comprehensive understanding of dauer larvae lifespan extension mechanisms and the generalization of those to other organisms, we need to push toward identifying the exact biological players of toxicity and recovery competition.

## Data Availability

The original contributions presented in the study are included in the article/Supplementary Material, further inquiries can be directed to the corresponding author.
