# Supplementary material for: Periodic ethanol supply as a path toward unlimited lifespan of Caenorhabditis elegans dauer larvae
Source: Front Aging. 2023 Sep 5;4:1031161. doi: 10.3389/fragi.2023.1031161 (PMC10507685; doi:10.3389/fragi.2023.1031161)
Supplement: Supplementary file 1 [file Presentation1.pdf]

Supplementary Material

1 NUMERICAL ALGORITHM AND MODEL PARAMETERS

The numerical simulations in this work use the Runge–Kutta methods of order four Lambert (1991). The parameters used in the model are given in Table 1. The value of j_{in} corresponds to the situation of no feeding and constant feeding.

$\tilde{k}_{1(\mathrm{daf2})}/\tilde{k}_4$	9	l_1/l_2	1.0
$\tilde{k}_{1(\mathrm{age1})}/\tilde{k}_4$	6.25	$j_{\mathrm{m}}/l_2\tilde{k}_4$	0.5
$\tilde{k}_{2(\mathrm{daf2})}l_2/\tilde{k}_4$	0.56	c_{h}/l_2	1.0
$\tilde{k}_{2(\mathrm{daf2aak2})}l_2/\tilde{k}_4$	$11,2$	$a(t=0)/l_2$	2.0
$k_3/\tilde{k}_4$	0.01	$l(t=0)/l_2$	42.0
l_{s}/l_2	80.0	$j_{\mathrm{in}}/l_2\tilde{k}_4$	0/0.9
$k_{\mathrm{d1}}/\tilde{k}_4$	12.0	$k_{\mathrm{d2}}/\tilde{k}_4$	3.75

Table S1. Parameters and initial conditions used in the simulation.

Initial conditions are motivated by experimental conditions. It is natural to consider the mitochondria activity to be unity since there is no damage at the beginning. Following similar reason the initial toxic level is set to zero. According to the simulations, the acetate level is a fast variable which converge to a certain balance value irrespective of the initial condition, and thus its initial value is not much relevant as long as it starts above the starvation limit. The initial lipid storage is determined similar to parameters in the model, where the value leading to the best fitting is taken.

The curves in Fig 8, are calculated by parameter scanning, were for each given pair of (ω_E, A) we run the simulation with a set of initial lipid storages equally spacing from low to high, and the "range" w corresponding to this pair of (ω_E, A) is given by the maximal value of w from the simulation result of this set of initial lipids. Scanning over initial lipid storage rules out the effect of initial conditions, as we want to explore the ability to have an unlimited lifespan irrespective of the initial conditions.

In total, the model contains 10 parameters and needs 4 initial values for the variables. To fix the parameter values we need to reproduce survival curves of 3 strains for 2 ethanol conditions (where only selected parameters are altered to recapitulate the mutant situation). In approximating the lifespan curves, we aimed to satisfy both the position of the characteristic lifetime (when survival drops to 50%) and the steepness of the survival curve decay. Additionally, we have certain biologically motivated limitations on the initial values and rates. While we have not performed a systematic scanning of the parameter space to identify suitable parameter sets, we suggest that the number of imposed constraints allowed us to converge to a unique set. Furthermore, for the case of periodic ethanol supply, we performed a proper sampling over the range of possible initial conditions to rule out the effect of the starting values.

2 DIVERGENCE OF THE LIFESPAN AT ONE SIDE

In Fig 7, there is a region of j_0 values considered to lead to an unlimited lifespan. When we slowly increase the value of j_0 across the lower boundary or decrease the value of j_0 across the higher boundary, the value of lifespan experiences a switch process from limited value to an unlimited one. This transition from limited to unlimited lifespan can be of two types: continuous divergence or a discontinuous jump. Here we briefly discuss which type the switch occurs for both the lower and higher boundaries. We first focus on the high ethanol boundary which we denote j_{re}.

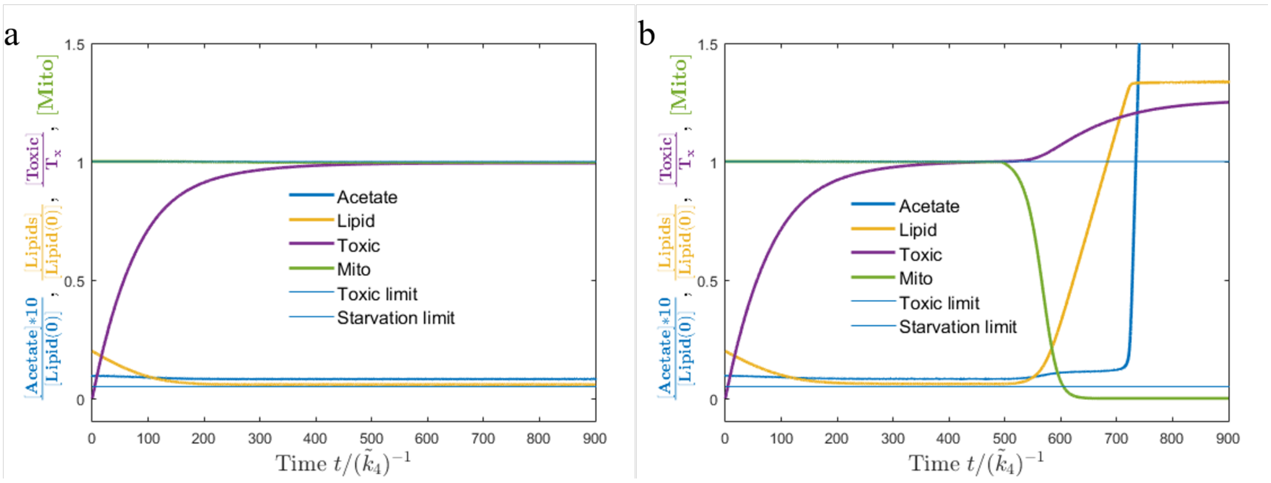

Figure S1. When $j_0 < j_{re}$, the toxic level grows and stays below its threshold value $c \lesssim c_h$ and the dauer survives (a). When $j_0 > j_{re}$ the toxic level grows higher than the threshold such that the dauer is damaged and dies (b).

The dynamics around but below the transition suggests that the trajectory $\bar{c}$ of the toxic compound c approaches a certain baseline value $\bar{c}_{ss}$ close to c_h exponentially.

$$(\bar{c}_{ss} - \bar{c}) = e^{-\kappa(t-t_{r0})}(\bar{c}_{ss} - c_{r0}) \tag{S1}$$

This approximation is supported by numerical simulations: the value of $\log(\bar{c}_{ss} - c)/c_h$ is shown in Figure 12.

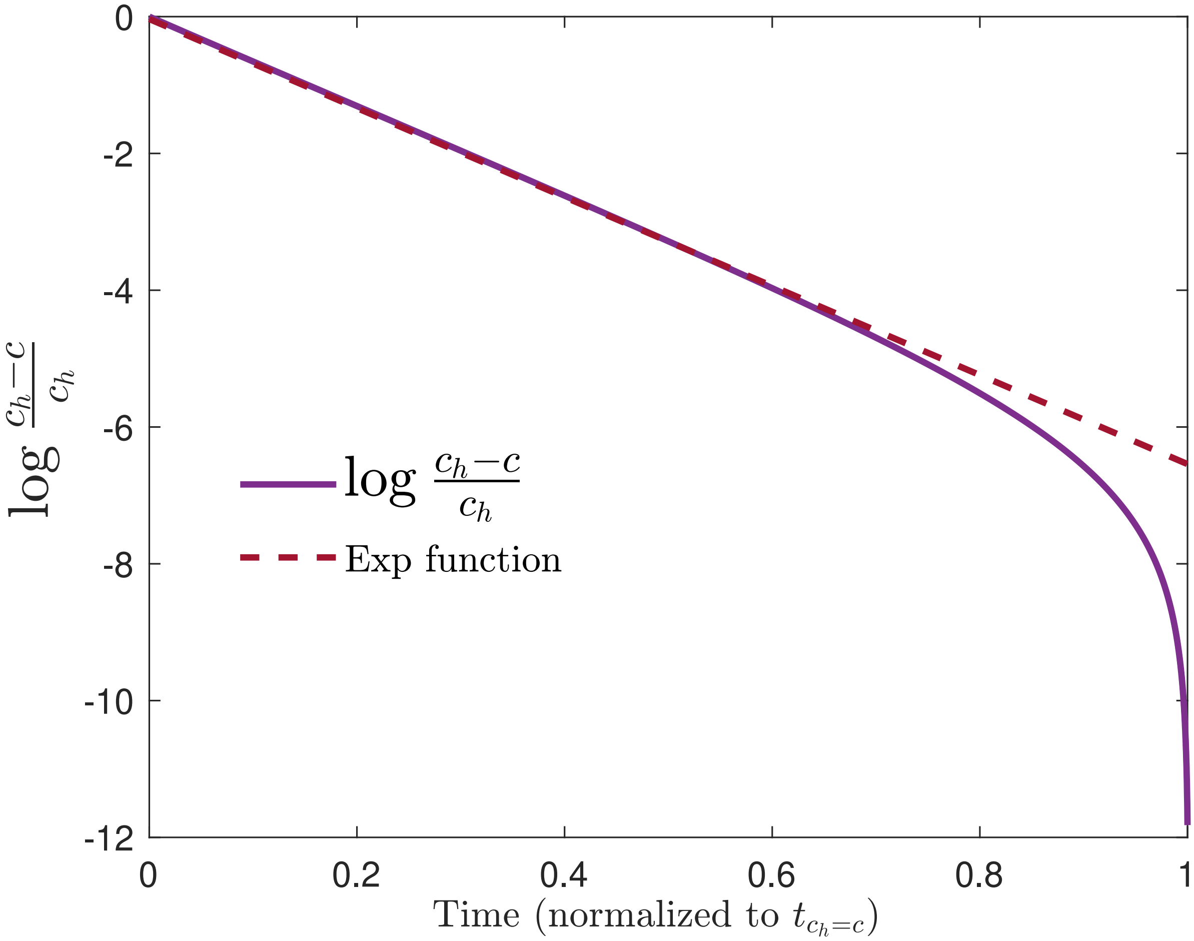

Figure S2. Around j_{re}, the trajectory of chemical compound c approaches the quasi-steady state $c_{ss} \leq c_h$ exponentially. c_h is the threshold value of toxic compound above which mitochondria start to damage.

Taking the logarithm of equation 9 yields a logarithmic divergence of time t for toxic compound to go across the threshold c_h. With considering all relevant parameters the expression of lifespan t_{ls} is thus:

$$t_{\mathrm{ls}} = t + t_{\mathrm{damage}} = -\frac{1}{\kappa}\log[\frac{(j_0 - j_{\mathrm{re}})}{\bar{c}_{\mathrm{ss}}}] + t_{\mathrm{damage}} \tag{S2}$$

where j_{re} is the transition point and κ is constants while t_{damage} is the time dauer could survive when irreversible damage happens. Numerical simulation of lifespan t_{ls} as function of the distance to boundary $j_0 - j_{re}$ is shown in Fig 12, which suggests that logarithmic function fits the functional form of divergence quite well.

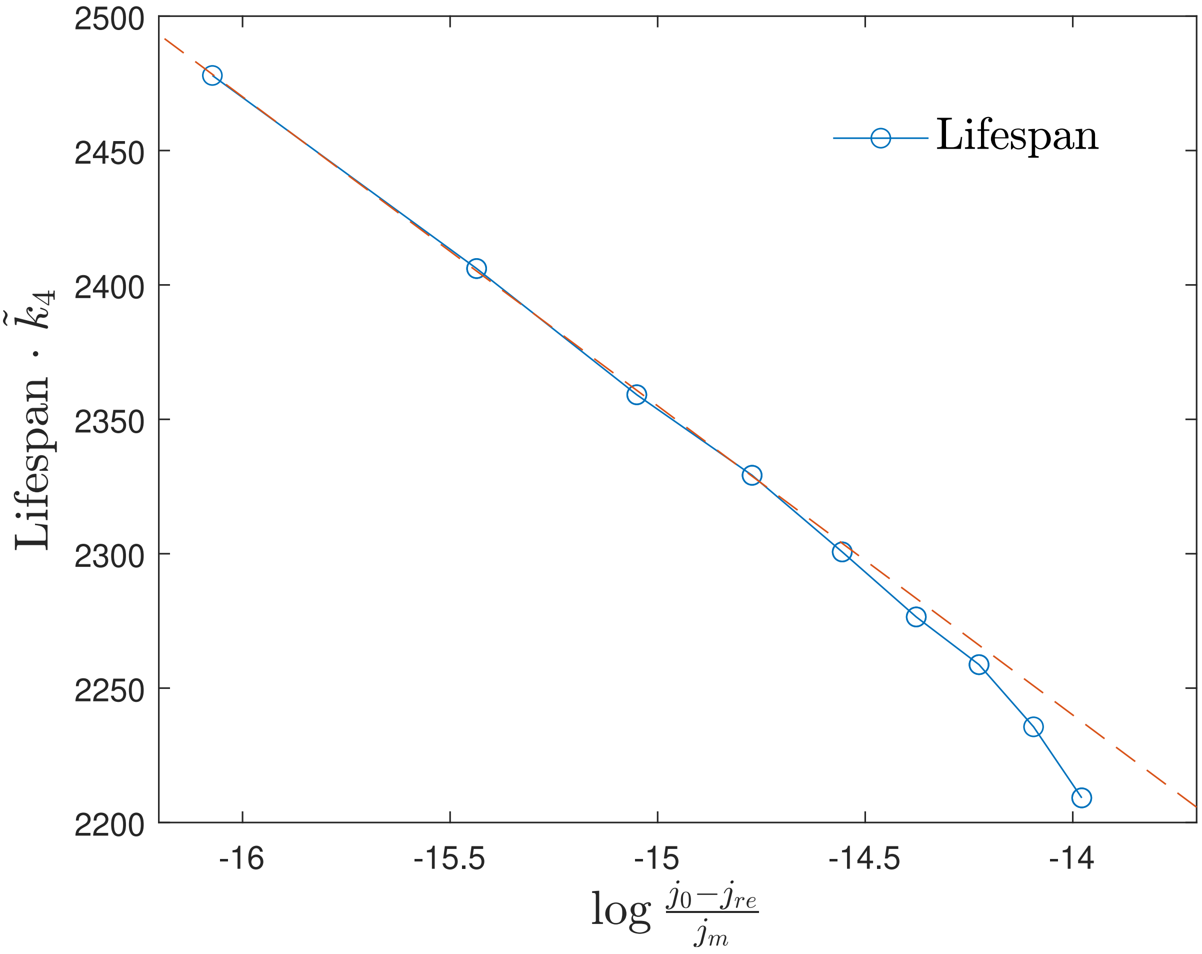

Figure S3. Logarithm fitting (red dashed line) of the divergence of lifespan at high ethanol edge. The factor $\frac{1}{\kappa}$ fits to $11.5/k_4$.

The switch process at the left edge j_{le} for small ethanol influx is challenging to treat analytically, but the numerical simulation up to precision limit indicate the switch process at j_{le} to be a discontinuous jump, see Fig S4.

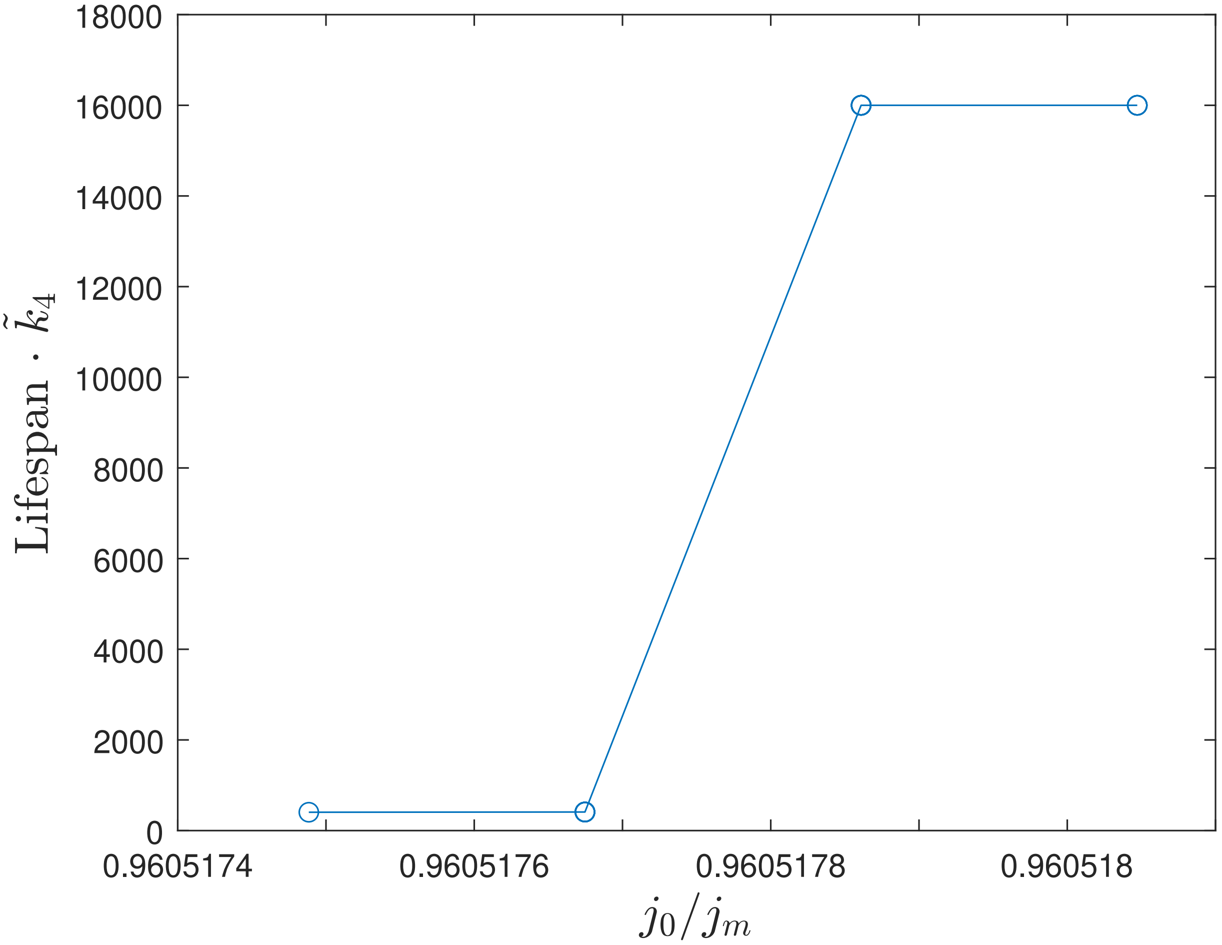

Figure S4. Jump of lifespan at low ethanol edge.

3 ANALYTICAL EXPLANATION OF THE DEPENDENCE OF RANGE ON PARAMETERS

Here we explain why $w(\omega_E, A)$ takes the functional form shown in Fig 8 through an analytical study. As discussed before, detoxification of the toxic compound at low acetate level and mitochondria regeneration at high acetate level must happen in alternating manner to allow for an unlimited lifespan.

Mathematically, in the state of the unlimited lifespan, the time dependent acetate level $a(t)$ should satisfy following relations, which corresponds to the level of acetate generating minimum “energy” flux j_{in}.

$$max[a] > j_m/k_4 \tag{S3}$$

$$min[a] < j_m/k_4, \tag{S4}$$

where the maximal and minimal are taken at large t when the initial condition is fully forgotten. In other words, the threshold value $a_{min} = j_m/k_4$ must be contained in the interval $(min[a], max[a])$, as shown in Fig 7. The range w is defined as the size of the interval where we can alter j_0 while keeping the lifespan of dauer to be unlimited. If we can estimate the dependence of $max[a]$, $min[a]$ on j_0, the above equations can be used to evaluate the range w. Here we assume that altering j_0 by a small constant δj will only change the value of $a(t)$ and thus its maximum and minimum in a linear way:

$$max[a](j_0 + \Delta j) = max[a](j_0) + \alpha\Delta j \tag{S5}$$

$$min[a](j_0 + \Delta j) = min[a](j_0) + \alpha\Delta j \tag{S6}$$

where α is the proportionality constant assumed to be the same for both $min[a]$ and $max[a]$. If we start with a j_0 such that equations S3 and S4 are satisfied, the value of δj we can choose such that equation S3 and S4 are still satisfied is given by:

$$max[a](j_0) + \alpha\Delta j > j_m/k_4 \tag{S7}$$

$$min[a](j_0) + \alpha\Delta j < j_m/k_4, \tag{S8}$$

$$\Delta j \in (\frac{1}{\alpha}[j_m/k_4 - max[a](j_0)], \frac{1}{\alpha}[j_m/k_4 - min[a](j_0)]) \tag{S9}$$

An estimated relation between “range” w and the acetate fluctuation region size $(max[a] - min[a])$ is thus given by:

$$\begin{aligned} w &\approx \frac{1}{\alpha}[j_m/k_4 - min[a](j_0)] - \frac{1}{\alpha}[j_m/k_4 - max[a](j_0)] \\ &\propto (max[a] - min[a]) \equiv 2A_{\text{acetate}} \end{aligned} \tag{S10}$$

where A_{acetate} defined in above equation is considered as the fluctuation amplitude of $a(t)$. Of course, that the profile of $a(t)$ does not vary with j_0 such that $min[a]$ and $max[a]$ share the same α is only a rough approximation. Numerical simulation estimating w and A_{acetate} for different feeding frequency ω_E suggests that equation S10 holds well when A_{acetate} is small, as shown in Fig S5.

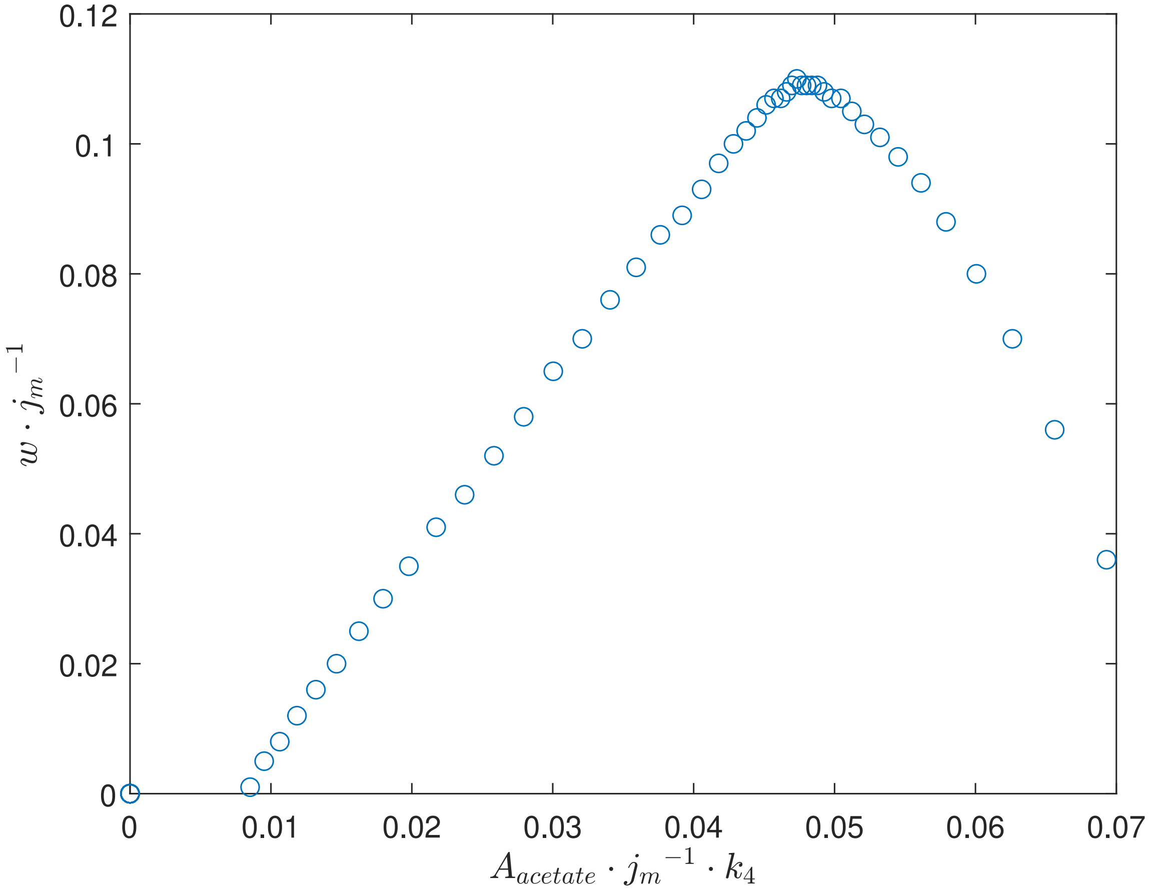

Figure S5. Width w as a function of acetate amplitude A_{acetate} for different feeding frequency ω_E with fixed A.

This region of small A_{acetate} and w corresponds to high feeding frequency (ω_{Et}) region according to Fig 8 and Fig 15. A linear analysis at high feeding frequency would help to further explain the relation between A_{acetate} and feeding parameters A and ω_{Et}.

Under sinusoidal feeding protocol with high frequency, it is observed that also a, l and c behave almost sinusoidally and oscillate around their average value:

$$a = \bar{a} + \delta a(t) \tag{S11}$$

$$l = \bar{l} + \delta l(t) \tag{S12}$$

$$c = \bar{c} + \delta c(t), \tag{S13}$$

The non-constant parameter k_1 in (6) and k_2 in (7) can be expanded as linear functions of l:

$$k_2 l \approx (\bar{k}_2 - \tilde{k}_2 \frac{1}{(l_2+\bar{l})^2}\delta l)(\bar{l}+\delta l) = \bar{k}_2\bar{l} + (\bar{k}_2 - \tilde{k}_2\frac{\bar{l}}{(l_2+\bar{l})^2})\delta l \tag{S14}$$

$$k_1 a \approx (\bar{k}_1 - \frac{\tilde{k}_1 l_1}{(l_1+l_s-\bar{l})^2}\delta l)(\bar{a}+\delta a) = \bar{k}_1\bar{a} + (\bar{k}_1\delta a - \frac{\tilde{k}_1 l_1 \bar{a}}{(l_1+l_s-\bar{l})^2}\delta l) \tag{S15}$$

The resulting equations are thus linear if we additionally assume there is no damage happening and $m = 1$. Numerical simulations suggest that the model with no damage approximation predicts similar behaviour of acetate oscillation amplitude A_{acetate} as in the original model (Fig S6): A_{acetate} decreases as we increase ω_E.

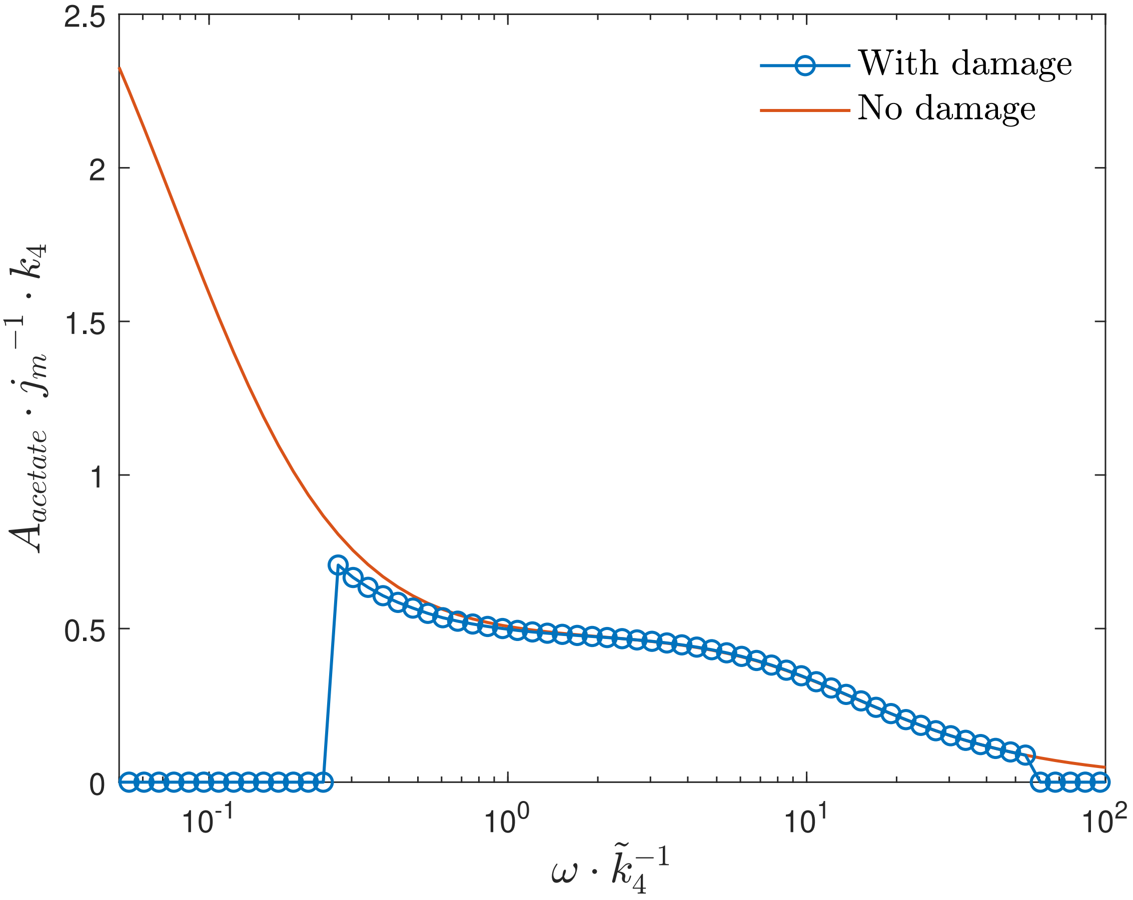

Figure S6. Difference between the original model and model with $m = 1$ is small at high frequency region defined as $\omega > k_4$.

The linearised model for δa, δl and δc thus reads:

$$\frac{d\delta a}{dt} = -\bar{k}_1\delta a + (\frac{\tilde{k}_1 l_1 \bar{a}}{(l_1+l_s-\bar{l})^2} + \bar{k}_2 - \tilde{k}_2\frac{\bar{l}}{(l_2+\bar{l})^2})\delta l + j_0 + Asin(\omega_E t + \phi) \tag{S16}$$

$$\frac{d\delta l}{dt} = \bar{k}_1\delta a - \frac{\tilde{k}_1 l_1 \bar{a}}{(l_1 + l_s - \bar{l})^2}\delta l - (\bar{k}_2 - \tilde{k}_2\frac{\bar{l}}{(l_2 + \bar{l})^2})\delta l \tag{S17}$$

$$\frac{d\delta c}{dt} = k_3(\bar{k}_2 - \tilde{k}_2\frac{\bar{l}}{(l_2 + \bar{l})^2})\delta l - k_c\delta c \tag{S18}$$

Since the equation for c is isolated from the other two equations of system under no damage ($m = 1$) assumption, the linearised model is essentially a two dimensional linear system driven by a sinusoidal force Strogatz (2000). The first two equations (S16) and (S17) can be decoupled and take the following form (ϕ is set to zero for convenience):

$$\frac{d\delta a}{dt} = -p_1\delta a + p_2\delta l + Asin(\omega_E t) \tag{S19}$$

$$\frac{d\delta l}{dt} = p_1\delta a - p_2\delta l. \tag{S20}$$

We reach the solution in general form:

$$\delta a = q_1 sin(\omega t) + q_2 cos(\omega t) \tag{S21}$$

$$\delta l = q_3 sin(\omega t) + q_4 cos(\omega t) \tag{S22}$$

to obtain:

$$q_1 = \frac{Ap_1}{(p_1 + p_2)^2 + \omega^2} \tag{S23}$$

$$q_2 = \frac{A(p_1 p_2 + p_2^2 + \omega^2)}{\omega[(p_1 + p_2)^2 + \omega^2]} \tag{S24}$$

$$q_3 = \frac{Ap_1}{(p_1 + p_2)^2 + \omega^2} \tag{S25}$$

$$q_4 = \frac{Ap_1(p_1 + p_2)}{\omega[(p_1 + p_2)^2 + \omega^2]} \tag{S26}$$

The corresponding oscillation amplitude of acetate and lipids is given by:

$$A_{\text{acetate}} = \sqrt{q_1^2 + q_2^2} \propto A \tag{S27}$$

$$A_{lipids} = \sqrt{q_3^2 + q_4^2} \propto A \tag{S28}$$

The consideration above suggests the proportionality relation between the oscillation amplitude of acetate and the feeding amplitude,

$$A_{\text{acetate}} \propto A, \tag{S29}$$

which agrees with the result shown in Fig 8. Equation S27 also describes the dependence of A_{acetate} as a function of ω_E, as shown in Fig S7. It is clear that the linear approximation captures the overall behaviour of A_{acetate} and thus the range w (according to Fig 14) as function of ω_E, but misses reproducing the exact functional form.

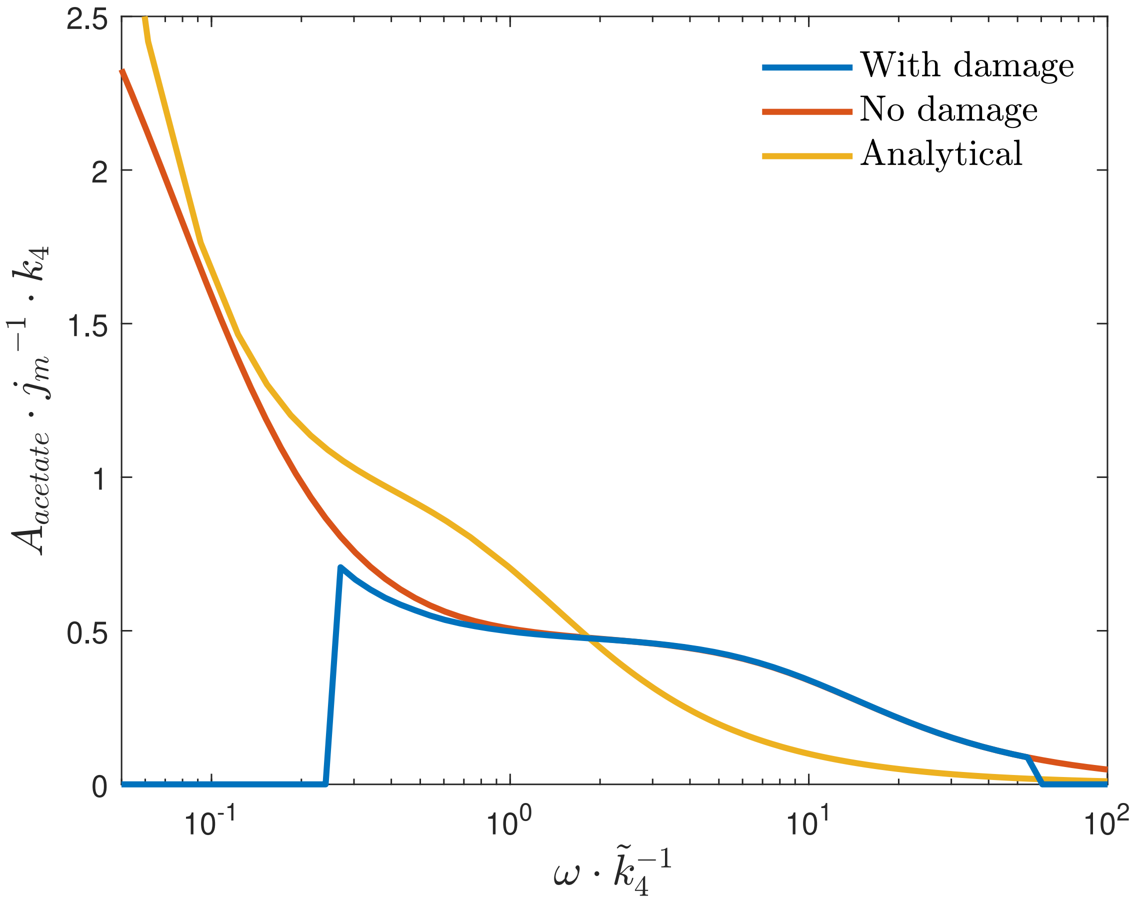

Figure S7. A_{acetate} vs. ω_{Et} of the original model (blue curve), no damage approximation (red curve) and analytical linear approximation (orange curve).

REFERENCES

Lambert, J. D. (1991). *Numerical Methods for Ordinary Differential Systems: The Initial Value Problem* (New York City: Wiley)

Strogatz, S. (2000). *Dynamics and Chaos* (New York City: Perseus Publishing)
